# An ecological animal model of subthreshold depression in adolescence: behavioral and resting state ^18^F-FDG PET imaging characterization

**DOI:** 10.1038/s41398-022-02119-1

**Published:** 2022-09-01

**Authors:** Georgine Accrombessi, Laurent Galineau, Clovis Tauber, Sophie Serrière, Esteban Moyer, Bruno Brizard, Anne-Marie Le Guisquet, Alexandre Surget, Catherine Belzung

**Affiliations:** UMR 1253, iBrain, Inserm, Université de Tours, CEDEX 1, 37032 Tours, France

**Keywords:** Depression, Predictive markers

## Abstract

The different depressive disorders that exist can take root at adolescence. For instance, some functional and structural changes in several brain regions have been observed from adolescence in subjects that display either high vulnerability to depressive symptoms or subthreshold depression. For instance, adolescents with depressive disorder have been shown to exhibit hyperactivity in hippocampus, amygdala and prefrontal cortex as well as volume reductions in hippocampus and amygdala (prefrontal cortex showing more variable results). However, no animal model of adolescent subthreshold depression has been developed so far. Our objective was to design an animal model of adolescent subthreshold depression and to characterize the neural changes associated to this phenotype. For this purpose, we used adolescent Swiss mice that were evaluated on 4 tests assessing cognitive abilities (Morris water maze), anhedonia (sucrose preference), anxiety (open-field) and stress-coping strategies (forced swim test) at postnatal day (PND) 28–35. In order to identify neural alterations associated to behavioral profiles, we assessed brain resting state metabolic activity in vivo using ^18^F-FDG PET imaging at PND 37. We selected three profiles of mice distinguished in a composite Z-score computed from performances in the behavioral tests: High, Intermediate and Low Depressive Risk (HDR, IDR and LDR). Compared to both IDR and LDR, HDR mice were characterized by passive stress-coping behaviors, low cognition and high anhedonia and anxiety and were associated with significant changes of ^18^F-FDG uptakes in several cortical and subcortical areas including prelimbic cortex, infralimbic cortex, nucleus accumbens, amygdala, periaqueductal gray and superior colliculus, all displaying higher metabolic activity, while only the thalamus was associated with lower metabolic activity (compared to IDR). LDR displayed an opposing behavioral phenotype and were associated with significant changes of ^18^F-FDG uptakes in the dorsal striatum and thalamus that both exhibited markedly lower metabolic activity in LDR. In conclusion, our study revealed changes in metabolic activities that can represent neural signatures for behavioral profiles predicting subthreshold depression at adolescence in a mouse model.

## Introduction

Depressive disorders affects all ages, including adolescents [1]. Adolescence requires personality adjustment; the slightest environmental alteration during this period of life can undermine the stability of the organism, increasing its vulnerability to affective disorders later in life or even inducing a subthreshold depressive state during adolescence. The onset of depressive disorders is frequent during adolescence [2] as at least one depressive episode is experienced by 15% of the 12–17-year-old population [3]. Generally, diagnosis of depressive disorders is not even detected in 40% of adolescents who are affected [4], although it represents a significant risk factor for suicide [5]. The symptomatic heterogeneity among patients has been identified as a critical cause that is able to hamper diagnosis [6].

At adolescence, some personality traits have been linked with higher risks of developing depressive symptoms [7–9] and correlate with severity of depressive symptoms [10, 11]. More specifically, negative affect and poor cognition increase vulnerability to depressive disorders in adolescence [12, 13] and high anxiety trait is a risk factor for the onset of adolescent and adult depressive disorders [14]. These traits are associated with negative affect, high trait anxiety and poor cognition as well as with altered activities of different brain areas like prefrontal cortex (PFC), amygdala, hippocampus and cerebellum in adolescents and adults [15–18].

Further, there are functional and structural modifications in the prefrontal and limbic areas that have been linked with the onset of adolescent depressive disorders including subthreshold depression [19–21]. It is noteworthy that these regions undergo neurochemical and physiological maturation during adolescence [22], which might underlie behavioral adjustments and promote the beginning of adolescent depressive disorders.

A high functional magnetic resonance imaging resting state activity was observed in the prefrontal cortex, the hippocampus, the thalamus, the striatum, and the amygdala in adolescents affected with different depressive disorders [19, 23–26]. There is a reduction in the volume of the hippocampus [27] which is intensified with the severity of depressive symptoms [28]. A decrease in the volume of the amygdala has also been found in adolescent with depressive symptoms [29]. Inconsistent results have been observed in prefrontal cortex with studies reporting higher volumes of the left dorsolateral prefrontal cortex in adolescents with depressive symptoms [22, 30], while others detected reduced volumes of the dorsolateral and medial prefrontal cortices of adolescent with subthreshold depression as well as of depressed adults [31–34].

Moreover, we observe a divergence of results for the prefrontal cortex like a greater volume of the dorsolateral and left-side prefrontal cortex in adolescent depressive disorders [22, 30] while adolescents with subthreshold depression [33] and major depression [32] as well as adult with depressive disorders show a significant atrophy of the dorsolateral and medial prefrontal cortex [31, 34].

Studies in human are however limited to investigate the neurobiological mechanisms when compared to animal models. Indeed, only animal models may enable approaches to precisely unravel the mechanistic underpinnings of disorders and vulnerability traits. Considering the lack of animal models for subthreshold depression at adolescence, our objective was to design such a model based on phenotypic profiles relevant for depressive disorders vulnerability in adolescent and to characterize the neural changes associated to these phenotypic profiles.

We used a mouse model as this species displays neuronal, hormonal, behavioral and developmental similarities with human subjects in adolescence [35, 36]. Adolescence is a life span that is determined by the transition from childhood to adulthood and is characterized by a myriad of physiological changes and behavioral disturbances that are associated with puberty [36]. In the rodent literature, this developmental stage correspond to the period starting at the end of weaning at PND 21 (Post Natal Day) and ending at PND 59 [37]. Most studies perform experimental manipulations in adolescence in order to increase depression-related behaviors at adulthood [38]. However, our study used a different approach as identification of depressive-like state was carried out using the spontaneous variation within a heterogeneous group of mice.

We used Swiss mice (300 mice) at early adolescence that were evaluated for their behavioral and cognitive abilities (PND 28–35) as well as for metabolic activity in basal conditions using ^18^F-FDG PET imaging (PND 37).

We used the Morris water maze for assessing spatial learning performances, the sucrose preference test for anhedonia, the forced swim test for stress-coping strategies and the Open field test for anxiety-like behaviors. A composite index was calculated to select mice for extreme in risk for depression discriminating high depression risk (HDR, 10% mice with low cognitive performance, anhedonia, passive stress-coping and anxiety), low depression risk (LDR, 10% mice with the opposite pattern) and intermediate depression risk (IDR, 10% mice with intermediate pattern). We then characterized the neural alterations associated to the different phenotypic profiles using of metabolic activity in basal conditions using ^18^F-FDG PET brain imaging (PND 37).

## Materials and methods

### Animals

A total number of 300 male Swiss mice was obtained from Janvier Labs (Le Genest-Saint-Isle, France) at the age of 3 weeks. They were familiarized with the animal facility for a week before the start of the experiment and kept under standard laboratory conditions (12-hour light-dark cycle, lights on at 8:30 p.m., 21 ± 2 °C, food, and water ad libitum, housed 4–5 per cage with plastic tunnels and shelters). All procedures were carried out according to European Directive 2010/63/EU guidelines on animal ethics, complied with the 3Rs and were approved by a local ethical committee and the French Ministry of Higher Education, Research and Innovation (#2019020412052173).

### Experimental design

**Fig. 1:**
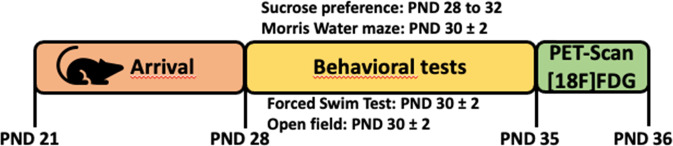
Schematic design of the experiment. mice were phenotyped using open field, sucrose preference, morris water and forced swim test (*N* = 300) at PND (Post Natal day) 28–35. Then, they were subjected to resting state ^18^F-FDG imaging (*N* = 90) at PND 37.

Animals were phenotyped (investigator blinded) between PND 28 and PND 35 using a test battery that included: open-field, sucrose preference, Morris’s water maze and forced swim test. They were then submitted to PET imaging using ^18^F-FDG in basal conditions at PND 37 (Fig. 1). Over 300 animals, mice were selected based on a composite z-score calculated from performances in the 4 different behavioral tests (see next section). Mice were thereafter assigned to one of the 3 experimental groups (HDR, IDR, LDR), each group encompassing 10% of the total mouse number (*n* = 30 per group). Sample size was calculated to detect an effect size of η² = 0.1 for the behavioral tests between the selected 3 groups at α = 0.05 and a statistical power (1–β) = 0.80. Testing was performed in a semi random order so that animals from all groups were tested each day, in each cohort.

### Behavioral phenotypes

#### Open field

The open field test takes advantage of the natural aversion of mice to large and unknown environments to evaluate the level of anxiety-related behaviors [39]. We used a circular arena with a diameter of 40 centimeters and delimitated several zones from the center to the periphery. We used 40 lx of lighting to illuminate the center of the device. Mice were placed individually close to the wall of the arena and the time in center zone (10 centimeters in diameter) was recorded during 10 min using and Ethovision a computerized videotracking system (Ethovision XT, 14.0, Noldus IT, The Netherlands).

#### Sucrose preference

This test was performed to assess motivation for a palatable stimulus: 1% sucrose solution. A low preference for sucrose (versus water) is commonly interpreted as anhedonia in mouse models of depression [40]. All the mice were individually housed during the five days of this test and had free access to two bottles all along: a first bottle with water and a second bottle with either water or 1% sucrose solution. The test started with a first stage of habituation for three days, which consisted in a first day where mice were exposed to two bottles of water followed by a second day where the bottle position was inverted, and eventually by a third day where mice were exposed to both a bottle of water and a bottle of 1% sucrose. Then, a final stage assessing the sucrose preference occurred the last two days of the test and consisted in measuring the consumption of both water and 1% sucrose solution for 2 days. The bottle position were interchanged between the 2 days. The consumption was estimated by measuring the weight difference of each bottle in 24 h each day. From this, we assessed the sucrose preference according to the following formula: 1% sucrose solution consumption / (1% sucrose solution + water) consumption.

#### Morris water maze

To assess cognitive functions, we used a modified version of the Morris water maze that can evaluate mouse performance for spatial learning and memory [41]. The device consisted in a pool (diameter: 90 cm) filled with water (22 °C), which also included a small platform (5 cm × 5 cm) immerged 1 cm below the water surface. We used 10 lx of lighting to illuminate the test room. The water was made opaque by adding small blue plastic lenses (length 2 mm). Many visual cues were present in the room, including furnitures (table, lamp) and a poster (white and black stripes) on the wall. First, a learning stage was performed consisting in 4 sessions of 3 trials, where the mouse had to swim to find the platform. If a mouse failed to find the platform in a trial of 1 min, the observer gently guided it to the platform. The 3 trials had different starting points within the same session with 1 min of rest on the platform for mice between each trial. After the last session of the learning stage, a probe test took place during which we removed the platform. The mouse was then allowed to swim for 1 min, and the frequency of crossing the initial position of the platform was recorded using a camera placed above the center of pool and analyzed using Ethovision (Noldus).

#### Forced swim test (FST)

This test allowed us assessing stress-coping behavioral strategy by placing the animals in a small water tank with neither exit nor platform forcing the mouse to keep swimming [42]. Swimming and struggling are commonly interpreted as active stress-coping behaviors while immobility is interpreted as passive stress-coping or despair-like behaviors. Mice were individually placed in a plexiglass cylinder (height: 40 cm; diameter: 18 cm) containing 15 cm of water (22 °C) for 6 min. We recorded and scored the time spent immobile during the 6 min of the test using Ethovision. The test was carried out under red light.

#### Z-score computation

To select mice with extreme multidimensional phenotypic traits at adolescence, we implemented a composite z-score (z_tot_) reducing in a single readout the depression vulnerability or level based on the mouse performances in 4 behavioral tests, each one assessing different phenotypic dimensions: anxiety-like behaviors (open field test), reward valuation (sucrose preference), cognitive performance (Morris water maze) and stress-coping strategy (forced swim test). Similar approaches have already been used and validated previously in animal models to assess integrated behavioral dimensions including emotionality in the context of animal models of chronic stress and depression [43].

z-scores are dimensionless standardized (=normalized) values indicating how many standard deviations a particular observation is below or above the mean of a reference group. In our study, we computed z-scores for each individual behavioral test per animal. Considering that successive cohorts of mice (*n* = 30 per cohort, 10 cohorts) were used all along the experiment for a total number of 300 mice, the reference group for the z-score computation of each mouse were its cohort group (*n* = 30). Accordingly, the z-scores obtained for each mouse were normalized against the mean and the standard deviation of its own cohort group in the corresponding test.

The z-scores of each individual test were therefore computed as such:

- z-score in the open-field (z_OF_) was calculated for each animal using normalization of its “duration passed in the central area of the OF arena” (against its cohort group),
- z-score in the sucrose solution preference (z_SP_)was calculated for each animal using normalization of its the “sucrose preference” (against its cohort group),
- z-score in the Morris water maze (z_WM_) was calculated for each animal using normalization og its “number of crossingthe platform position during the probe test” (against its cohort group).

z-score in the FST (z_FST_) was calculated for each animal using normalization of its “duration passed immobile during the 6-min FST” (against its cohort group).The z_tot_ score calculation was constructed in a way that (1) a low negative z_tot_ score reflects a higher depression vulnerability or level, and that (2) a high positive z_tot_ score reflects a lower depression vulnerability or level, With this purpose, the z_tot_ score was computed according to the following formula:

$$z_{tot} = \frac{{z_{OF} + z_{SP} + z_{WM} - z_{FST}}}{4}$$

Indeed, the directionality of the z-scores from each individual test within the z_tot_ formula was adjusted in order to have a positive z_tot_ when the animal displayed a lack of depression vulnerability or levels. Hence, the formula associate the sign “+” to z_OF_, z_SP_ and z_WM_ and the sign “−“ to z_FST_, denoting high center exploration in OF test, high sucrose preference, high platform position crossings in the water maze, and/or low immobility duration in the forced swim stress, reflecting together low or lack of depression vulnerability or level.

Eventually, the individual test z-scores and the z_tot_ score of each mouse were computed within each cohort. The mice within each cohort having the 10% lowest (*n* = 3 per cohort, 30 in total), 10% intermediate (*n* = 3 per cohort, 30 in total) and 10% highest (*n* = 3 per cohort, 30 in total) z_tot_ scores were selected to make up HDR, IDR and LDR groups respectively. HDR are the mice with higher probability to exhibit low time percentage in the center of the open field, poor sucrose preference, low frequency at the platform location in the Morris water maze, high immobility duration in the FST; LDR mice having higher probability to exhibit high time percentage in the center of the open field, high sucrose preference, high frequency at the platform location in the Morris water maze and low immobility duration in the FST; and IDR mice having intermediate scores between LDR and HDR.

#### Brain imaging

Metabolic imaging using ^18^F-FDG was performed under basal conditions. Mice were habituated to the PET experimental procedures for 3 days and were not fasted before the scan. At PND 37, the day of brain-imaging acquisition, awake mice were injected with ^18^F-FDG (18.5 MBq/100 g i.p.; Cyclopharma, Tours, France), and placed in their home cage for 45 min. Then, animals were anesthetized using isoflurane 4% (Baxter, Maurepas, France), placed on a heating pad (Minerve, Esternay, France) and centered in the field of view of the Explore VISTA-CT microPET camera (GE Healthcare, Velizy, France). A CT-scan was performed for attenuation correction of PET images and a list-mode PET acquisition of 30 min started 60 min after ^18^F-FDG injection. After data reconstruction using a 2-D OSEM algorithm, all images were co-registered and normalized for tissue activity in the whole brain. Quantitative results were expressed as mean ± SD and were presented on Z-score maps using an array of regions of interest already defined in PMOD v3.2 software (PMOD Technologies Ltd, Switzerland).

During the experiments, the respiratory rate and body temperature of each animal were monitored and kept as constant as possible (70 respirations per minutes and 37 °C, respectively). List-mode scans were rebinned into 6 frames of 300 s, corrected for random, scatter and attenuation, and images were reconstructed using a 2-D OSEM algorithm (GE Healthcare, Velizy, France) into voxels of 0.3875 × 0.3875 × 0.775 mm^3^. Data summed over the entire acquisition were used for image registration. Since brain anatomy is very similar for mice of similar weight, registration was accomplished as a rigid body transformation, with no warping or scaling. Each summed scan was individually smoothed with a Gaussian filter to improve the signal-to-noise ratio and to reduce the bias of misregistration into template space. For this smoothing, a kernel of 0.6 × 0.6 × 0.6 mm3 FWHM was used. Each scan was coregistered using PMOD v3.2 software (PMOD Technologies Ltd, Switzerland) to a ^18^F-FDG PET template in Paxinos coordinates [44] using a mutual information similarity function with Powell’s convergence optimization method [45]. The results were visually checked for misregistration. Each summed image was also used for statistical analysis. The regions of interest (ROI) atlas of Mirrione in Paxinos coordinates were merged to create a whole brain mask (WBM). To normalize the ^18^F-FDG uptake, tissue activity was divided by the whole brain activity, calculated as the average activity in the WBM. Prior to statistical analysis, the WBM was applied over all PET scans to exclude extracerebral regions.

#### Statistical analysis

Behavioral data were analyzed from Z-score computation and with one-way analysis of variance (ANOVA). The Tukey’s test was used for post hoc when the F value was significant (*P* < 0.05). All results are presented as mean ± SEM in the figures and mean ± SD in the text.

For PET imaging, a voxel-based analysis was used to assess the differences in cerebral ^18^F-FDG uptake between the averaged brains of HDR vs LDR, HDR vs IDR and LDR vs IDR groups. This was performed using unpaired Student’s two-tailed *t*-test with *p*-values corrected for multiple comparisons using the Benjamini-Hochberg control of false discovery rate [46]. However, all the individual voxel comparisons missed significance, as described in other PET studies with low degrees of freedom [47]. Therefore, Z-score maps with a threshold of *p* = 0.05 for uncorrected *p* values were generated with signals extracted from regions of interest for further analysis when representing at least 50 contiguous voxels. Effect size was evaluated for each significant differences observed and expressed as *d* values corresponding to a moderate, large or very large effect sizes for *d* values inferior to 0.80, compared between 0.80 and 1.20 or superior to 1.20, respectively [48, 49].

## Results

### Z-score computation

**Fig. 2:**
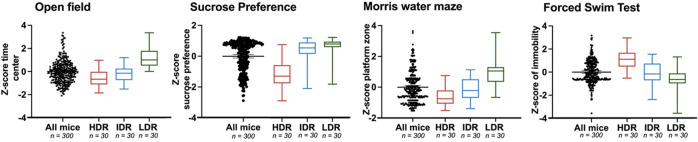
Spontaneous vulnerability at adolescence (*N* = 300) between PND 29–34. After Z-score computation, we selected 10% of extreme and intermediate phenotype. HDR high depression risk, IDR intermediate depression risk, LDR low depression risk, PND post natal day.

To select mice with extreme multidimensional phenotypic traits at adolescence relevant to subthreshold depression, we employed a composite z-score (z_tot_) reducing in a single readout the mouse performances across tests for anxiety-like behaviors (open-field), reward valuation (sucrose preference), spatial learning and memory (Morris water maze), and stress-coping strategy (FST) using spontaneous interindividual variability in swiss mice (*n* = 300). From this analysis, we selected the most extreme 10% mice for z_tot_ as well as the 10% intermediate mice, distinguishing each phenotype group: HDR, IDR and LDR (Fig. 2).

### Behavioral phenotypes

#### Open field

**Fig. 3:**
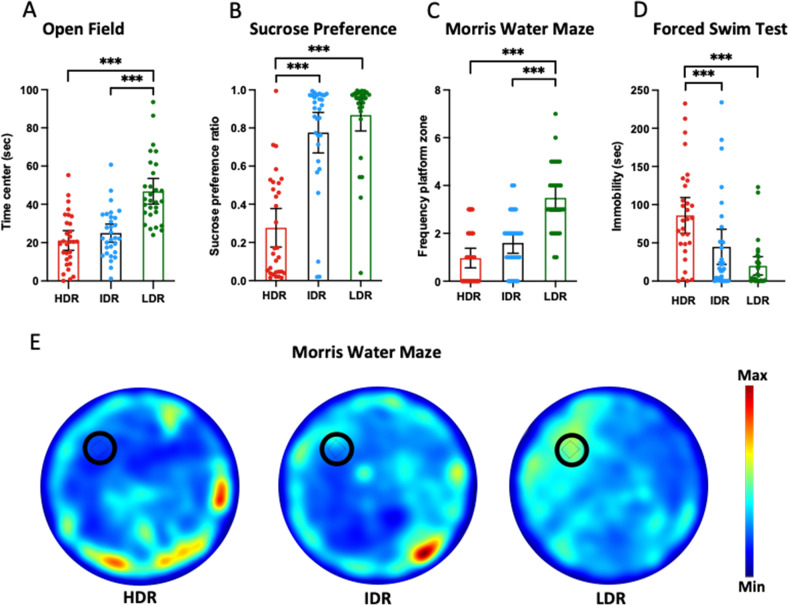
Behavioral profile after Z-score computation (*n* = 90). **A** Open field (anxiety). **B** Sucrose preference (anhedonia). **C** Morris water maze test (cognition). **D** Forced swim test (resignation). **E** Representative heatmap images of platform zone frequency for each phenotype in Morris water maze. Results are presented as mean ± S.E.M. ***P < 0.001 between groups. PND post natal day, HDR high depression risk, IDR intermediate depression risk, LDR low depression risk.

To characterize anxiety-like behaviors, all mice (*n* = 300) were subjected to an open field test at PND 30 ± 2 (Fig. 2). After Z-score calculation, the subdivision of mice allowed us observing the behavioral profiles of the selected phenotypes. A one-way ANOVA and post hoc Tukey’s test compared the time spent in the center of the open field and revealed a significant effect (one-way ANOVA, F_2,87_ = 26, *P* < 0.0001). LDR mice (46.81 ± 17.70) spent more time in the central area compared to HDR (21.13 ± 13.75) and IDR (24.96 ± 12.69) (Fig. 3A).

#### Sucrose preference

Mice (*n* = 300) were subjected to this test from PND 29 to PND 34 (Fig. 2). After Z-score calculation, we observed a significant difference between the different phenotypes (one-way ANOVA, F_2,87_ = 44, *P* < 0.0001) indicating that HDR mice (0.27 ± 0.27) show reduced sucrose preference ratio when compared to IDR (0.77 ± 0.28) and LDR (0.86 ± 0.22) (Fig. 3B).

#### Morris water maze

We assessed the spatial memory of all mice (*n* = 300) in the probe test at PND 30 ± 2 (Fig. 2). After Z-score calculation, we found that LDR mice (3.48 ± 1.43) displayed a high frequency at crossing the location of the platform compared to IDR (1.60 ± 1.16) and HDR (0.96 ± 1.09) (one-way ANOVA, F_2,87_ = 33, *P* < 0.0001; Fig. 3C).

#### Forced swim test

All mice (*n* = 300) underwent this test between at PND 30 ± 2 (Fig. 2). After Z-score calculation, the HDR mice (85.85 ± 62.61) showed a significantly higher time of immobility than IDR (44.81 ± 61.53) and HDR (19.91 ± 31.56) mice (one-way ANOVA, F_2,87_ = 11, *P* < 0.0001; Fig. 3D).

### Brain imaging

#### Brain Metabolic Activity in HDR vs IDR mice

**Fig. 4:**
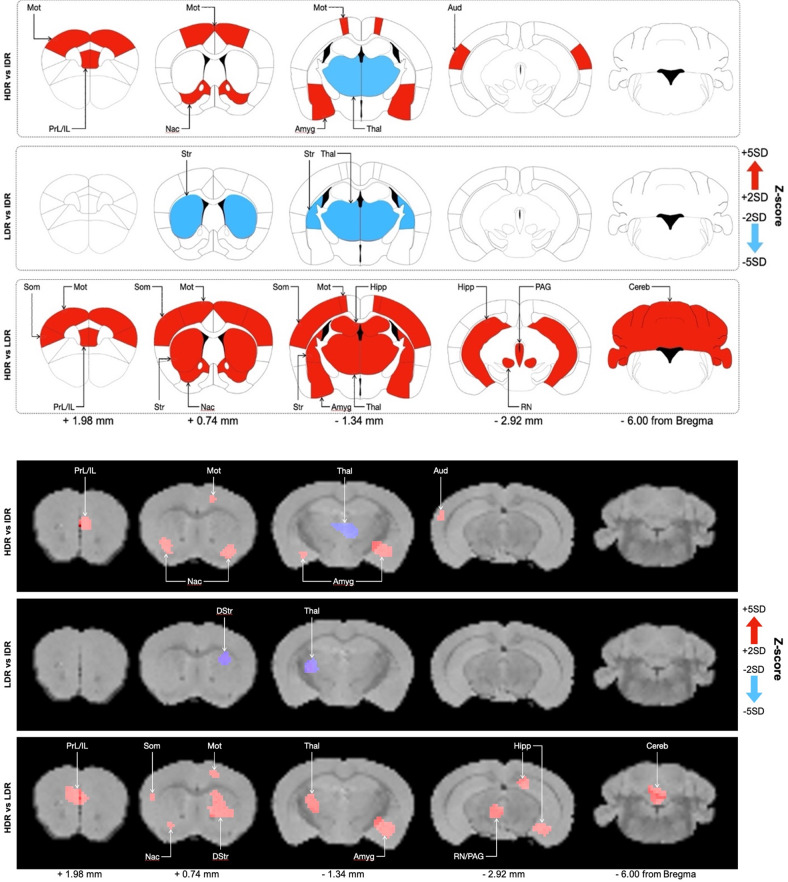
Comparisons of the changes in metabolic activity in the different phenotypes at PND 37. **A** Summary of the significant increases (red) and decreases (blue) in uptake observed in HDR vs LDR; HDR vs IDR and IDR vs LDR (*n* = 33) at PND 37 presented on representative coronal plates of the Paxinos and Watson atlas with *P* < 0.01. **B** Example of the significant differences in ^18^F-FDG uptake observed in HDR vs LDR; HDR vs IDR and IDR vs LDR (*n* = 33) at PND 37 presented on coronal images of Z-scores maps fused with an MRI template (increases in 18-FDG uptake in red, decreases in ^18^F-FDG uptake in blue). Aud dorsal secondary auditory cortex, Thal thalamus, Amyg amygdala, Hipp hippocampus, PAG periaqueductal gray, RN red nucleus, DStr dorsal striatum, Str striatum, IL infralimbic cortex, PrL prelimbic cortex, Mot motor cortex, Som somatosensory cortex, Nac nucleus accumbens, cereb cerebellum, PND post natal day, HDR high depression risk, IDR intermediate depression risk, LDR low depression risk.

At PND 37, in HDR mice the metabolic activity of the infralimbic and prelimbic (Z = 2.60 ± 0.38; *P* = 0.0047), dorsal secondary auditory (Z = 2.27 ± 0.14; *P* = 0.0026) and motor cortex (Z = 2.27 ± 0.16; *P* = 0.0071), ventral striatum (nucleus accumbens) (Z = 2.58 ± 0.43; *P* = 0.0067), amygdala (Z = 2.61 ± 0.46; *P* = 0.0028), was significantly increased compared to IDR mice. On the other hand, the thalamus (Z = − 2.48 ± 0.32; *P* = 0.0047) of HDR mice shows a hypoactive metabolism compared to the one of IDR mice (Fig. 4, Table 1). There was no difference regarding the other brain regions.

**Table 1:** Statistical significances between the different phenotypes.

	HDR vs IDR			LDR vs IDR			HDR vs LDR		
	Z-Score	*d* value	*p* value	Z-Score	*d* value	*p* value	Z-Score	*d* value	*p* value
Cortex									
OF	–	–	–	–	–	–	–	–	–
PrL/IL	2.60 ± 0.38	0.98 ± 0.10	*p* = 0.0047	–	–	–	2.59 ± 0.35	0.99 ± 0.10	*p* < 0.0001
Cing	–	–	–	–	–	–	–	–	–
Ins	-	–	–	–	–	–	–	–	–
Mot	2.27 ± 0.16	0.89 ± 0.05	*p* = 0.0071	–	–	–	2.36 ± 0.20	0.93 ± 0.06	*p* < 0.0001
Som				–	–	–	2.53 ± 0.34	0.98 ± 0.10	*p* < 0.0001
Par	–	–	–	–	–	–	–	–	–
RSC	–	–	–	–	–	–	–	–	–
Aud	2.27 ± 0.14	0.89 ± 0.04	*p* = 0.0026	–	–	–	–	–	–
Vis	–	–	–	–	–	–	–	–	–
Temp	–	–	–	–	–	–	–	–	–
Subcortical areas									
DStr	–	–	–	−2.31 ± 0.17	0.88 ± 0.05	*p* = 0.0045	2.57 ± 0.38	0.99 ± 0.10	*p* < 0.0001
VStr	2.58 ± 0.43	0.97 ± 0.12	*p* = 0.0067	–	–	–	2.26 ± 0.11	0.90 ± 0.03	*p* = 0.0002
Thal	−2.48 ± 0.32	0.95 ± 0.08	*p* = 0.0047	−2.50 ± 0.38	0.93 ± 0.11	*p* = 0.0022	2.38 ± 0.28	0.93 ± 0.08	*p* < 0.0001
Amyg	2.61 ± 0.46	0.98 ± 0.12	*p* = 0.0028	–	–	–	2.60 ± 0.34	1.00 ± 0.10	*p* < 0.0001
Hypo	–	–	–	–	–	–	–	–	–
Hipp	–	–	–	–	–	–	2.36 ± 0.23	0.93 ± 0.07	*p* < 0.0001
PAG	–	–	–	–	–	–	2.46 ± 0.29	0.96 ± 0.08	*p* < 0.0001
SupCol	–	–	–	–	–	–	2.71 ± 0.50	1.02 ± 0.13	*p* < 0.0001
RN	–	–	–	–	–	–	2.96 ± 0.61	1.08 ± 0.15	*p* < 0.0001
Cerebellum	–	–		–	–	–	2.35 ± 0.21	0.93 ± 0.06	*p* < 0.0001

*OF* orbitofrontal cortex, *Cing* cingulate cortex, *Ins* insular, *Par* parabrachial nucleus, *RSC* retrosplenial cortex, *Vis* visual cortex, *Temp* temporal cortex, *Hypo* hypothalamus, *Aud* dorsal secondary auditory cortex, *Thal* thalamus, *Amyg* amygdala, *Hipp* hippocampus, *PAG* periaqueductal gray, *RN* red nucleus, *DStr* dorsal striatum, *VStr* ventral striatum, *IL* infralimbic cortex, *PrL* prelimbic cortex, *Mot* motor cortex, *Som* somatosensory cortex, *Nac* nucleus accumbens, *cereb* cerebellum, *SupCol* superior colliculus, *HDR* high depression risk, *IDR* intermediate depression risk, *LDR* low depression risk.

#### Brain metabolic activity in LDR vs IDR mice

At PND 37, in the striatum (Z = −2.31 ± 0.17; *P* = 0.0045) and thalamus (*Z* = −2.50 ± 0.38; *P* = 0.0022) the [18F]DG uptake was increased in IDR compared to LDR mice (Fig. 4, Table 1). No difference was seen between these groups regarding the other brain regions.

#### Brain metabolic activity in HDR vs LDR mice

At PND 37, ^18^F-FDG uptake was increased in HDR mice compared to LDR mice in the infralimbic and prelimbic (Z = 2.59 ± 0.35; *P* < 0,0001), somatosensory (Z = 2.53 ± 0.34; *P* < 0,0001), and motor cortex (Z = 2.36 ± 0.20; *P* < 0,0001), in the ventral (nucleus accumbens) (Z = 2.26 ± 0.11; *P* = 0.0002), and dorsal striatum (Z = 2.57 ± 0,38; *P* < 0,0001), thalamus (Z = 2.38 ± 0.28; *P* < 0,0001), amygdala (Z = 2.60 ± 0.34; *P* < 0,0001), hippocampus (Z = 2.36 ± 0.23; *P* < 0.0001), cerebellum (Z = 2.35 ± 0.21; *P* < 0.0001), periaqueductal gray (Z = 2.46 ± 0.29; *P* < 0.0001) and red nucleus (Z = 2.96 ± 0.61; *P* < 0.0001) (Fig. 4, Table 1). The metabolic activity remained unchanged in the other brain regions.

## Discussion

With the objective to characterize mice with varying depression risk at adolescence, we identified 3 profiles of mice based on their spontaneous interindividual variability in behaviors: the HDR, IDR, and LDR mice. The HDR mice were selected for a phenotype displaying decreased cognitive performance, anhedonia, elevated anxiety-like behaviors and passive stress-coping strategy, which was associated with changes in metabolic activity of brain regions playing a key role in depressive-like behaviors such as amygdala and nucleus accumbens. Based on the construction of our selection strategy, LDR mice displayed the opposite phenotype, exhibiting lower anxiety, anhedonia, and despair in addition to a better performance in spatial memory as well as a low level of changes in metabolic activity of the regions as given above. We present that spontaneous interindividual variability in adolescent Swiss mice generate a panel of changes in metabolic activity based on different behavioral phenotypes.

We propose that HDR mice may represent a rodent model of depression risk or subthreshold depression at adolescence. Indeed, human adolescents with higher risk for adolescent depressive disorders show high trait anxiety and consummatory anhedonia [14, 50]. In the same vein, individuals that present higher anxiety and vulnerability to depressive disorders are prone to present disrupted cognitive abilities including altered acquisitions in spatial learning tasks [51].

These results are also convergent with animal studies showing that these different behavioral dimensions can be associated one with the others. For example, rats with increased immobility in forced swim test showed poor performance in spatial memory tasks [52]. Accordingly, our selection of mice for extreme in multidimensional phenotypic traits at adolescence are consistent with behavioral traits associated with distinct vulnerability to depressive disorders as showed by previous preclinical and clinical studies [53–55].

Our study identified divergent patterns of brain resting state metabolic activity according to the phenotypic category. Indeed, our results revealed that the resting state metabolic activity in the prefrontal cortex (infralimbic and prelimbic cortex), the motor and somatosensory cortex, the periaqueductal gray, the red nucleus, the striatum, the nucleus accumbens, the hippocampus, the amygdala, the thalamus and the cerebellum were significantly more elevated in HDR compared to LDR. When we compared the HDR to the IDR, a significant hyperactivity was found in all these regions except in the thalamus that displayed a hypoactivity instead. The metabolic activity of the LDR and the IDR remained close except for the thalamus and the striatum that showed lower activity in LDR. The metabolic profile of the HDR is very close to other findings from neuroimaging studies in human subjects with subthreshold depression at adolescence. Indeed, an hyperactivation at resting state was found in the prefrontal cortex, the striatum, the thalamus and the hippocampus of depressed adults [19, 23, 25] and depressed adolescents [26] as revealed by PET and functional magnetic resonance imaging (fMRI). Further, using fMRI at resting state and emotional information processing, it was shown that activity was increased in the nucleus accumbens and amygdala of adolescents with depressive disorders and anxiety trait [56–59], as well as in depressed adults [2, 59, 60]. Another evidence suggesting a link between the brain activity of the cerebellum and depressive disorders through fMRI has been observed in individuals with subthreshold depression, which displayed an overactivation of cerebellum [61]. Finally, in an adult animal model of depressive-like behaviors, the ^18^F-FDG uptake was found to be increased in the striatum and the cerebellum [62]. Taken together, these data confirm that the HDR mice can be proposed as an animal model of sub-threshold depression in adolescence.

However, it is worth to mention that our results also differ from some studies that found a reduced neuronal response in the striatum and cerebellum of adolescents with subthreshold depression using resting state fMRI, [63, 64]. Further, some preclinical studies found a reduction in the metabolic activity of the hippocampus and periaqueductal gray in adults rodent as response to acute swim stress in association with behavioral despair involved in depression [62, 65]. Others found that areas like the prefrontal cortex, the thalamus, the amygdala, the hippocampus, the periaqueductal gray, the striatum, the nucleus accumbens, the cerebellum that are affected in adults depressive disorders were also impacted in adolescents depressive disorders in both human and animal studies [59], which is similar to our findings on vulnerability to depressive symptoms in adolescence. However, other findings differed from ours, particularly regarding the hippocampus, periaqueductal gray, striatum, and cerebellum which have been associated with a reduced activity in several reports while our study associated the vulnerability phenotype HDR with an increase activity in these areas. A possible explanation for this discrepancy is that neuroimaging is not performed at the same age in these other studies and/or because of species differences.

The changes of brain activity observed in our study in the HDR mice suggests that the behavioral phenotype observed in these mice might be related to these neural underpinnings. It is interesting to note that many regions engaged in these differences include cortico-limbic regions that have a profound influence on cognitive processing and emotional regulation [66]. Indeed, PFC, a pivotal brain center for the regulation of thought and behavior, is involved in cognitive and attentional control, as well as in the regulation of affect and the behavioral flexibility [2, 67, 68]. Deficits in these functions have been associated with vulnerability to depressive disorders [69, 70]. Further, passive stress-coping and despair-like behaviors have been associated with activity changes in the prefrontal cortex in mice [62] which is consistent with our findings showing that HDR mice displayed passive stress-coping behaviors in the FST together with metabolic hyperactivity in the prefrontal cortex. The nucleus accumbens participates in reward processing and its dysregulation contributes to symptoms of anhedonia in depression [71–73]. Studies using the social defeat paradigm in rodents demonstrated that mice exhibiting vulnerability to social defeat in the model or to develop depressive symptoms such as anhedonia, also show overactivation in the nucleus accumbens (eg: increased BDNF signaling, elevation of CREB activity [74–77]. These studies show that social defeat produces pathological effects (anhedonia) which can be explained by behavioral susceptibility to stress. In our case, this nucleus accumbens hyperactivity emerges in mice without manipulation (spontaneous phenotype) and exempt from stress paradigm seems to be free of causal link with the presence of anhedonia. However, this suggests that the disruption of this region in these HDR mice is associated with behavioral results.

The amygdala plays a key role in mood, emotional memory, fear and anxiety [71, 73, 78–82]. The high anxiety behavior found in HDR mice can thus be associated with metabolic hyperactivity of the amygdala. Interestingly, this link between anxiety and the increased activity of the amygdala has also been found in adolescent primates using functional neuroimaging [83]. Some functions like explicit memory, working memory, spatial learning and spatial memory rely on the hippocampus both in humans and in animal models of depression [72, 84–87]. Using fMRI, abnormal activity of the hippocampus has been observed together with memory impairment in patients with depressive disorders [19]. Here, the spatial memory deficit that we found related to HDR phenotype is associated with an increase in the metabolic activity of hippocampus. Further on, the thalamus, which participates in emotional salience [26], and the cerebellum, which is involved in emotional and cognitive processing in depressive disorders [63, 88], displayed altered metabolic activity in the HDR phenotype. Finally, the periaqueductal gray, the motor and somatosensory cortex and the red nucleus are also hyperactive in the HDR mice. Interestingly, activity of these brain regions is altered in adolescents with depressive symptoms or with high vulnerability to develop depressive symptoms and this can be normalized after antidepressant treatments with fluoxetine. It has thus been suggested to represent a marker of vulnerability to depressive disorders and of treatment response [59, 89].

Individual differences illustrated in our study are associated with anxiety-like behaviors, cognitive processing, stress-coping strategy and affect regulation, which influence each other and might be associated to distinct patterns of brain activity. Adolescent animals with the “high anxiety, cognitive impairment, passive stress-coping, negative affect” profile suggesting a subthreshold depression at adolescence could provide the ground for later development of this disorder at adulthood [82]. Similar profiles have also been found in human adolescents indicating the reliability of our spontaneous interindividual variability in adolescent mice [13]. More precisely, the “high anxiety, cognitive impairment, passive stress-coping, negative affect” profile of the HDR mice is similar to human traits of high neuroticism, low extraversion and low conscientiousness, which are proposed to be predictors of vulnerability to depressive disorders [79, 90]. Conversely, LDR individuals which display “low anxiety, good cognitive performance, active stress-coping, positive affect” might be proposed as a model of resilience to develop depressive disorders at adulthood, corresponding to medium neuroticism, medium extraversion, and high conscientiousness in human subjects [53, 90, 91]. In summary, the HDR mice can be considered as an animal model displaying the behavioral and neural markers associated to adolescent subthreshold depression or higher depression risk. This model will allow to design environmental and neuromodulation interventions to modify risk trajectories. However, some limits should also be highlighted, as our study does not allow to establish a causal relationship between the neural and the behavioral phenotype: longitudinal studies could be helpful to provide such causal link. As our study was set up using a large number of animals, we only included male animals and did not use female animals, which is a limitation. Further investigation should be done at adolescence using female mice.

## Conclusion

The combination of behavioral traits in adolescent mice allowed us sorting different profiles linked to the risk of developing depressive-like behaviors. These individual differences obtained in adolescence in anxiety, affect, cognition, stress-coping strategy and emotional reactivity in connection with the imbalance of neuronal activity between different brain regions suggest a subthreshold depression at adolescence. Selection of mice based on the spontaneous interindividual variability in adolescent mice thus represents a valid approach to model these features.
